# Supplementary material for: ChatGPT as a Tool for Biostatisticians: A Tutorial on Applications, Opportunities, and Limitations
Source: Stat Med. 2025 Oct 23;44(23-24):e70263. doi: 10.1002/sim.70263 (PMC12548020; doi:10.1002/sim.70263)
Supplement: Supplementary file 1 — Data S1. Supporting Information. [file SIM-44-0-s001.zip › Supplementary Materials Additional.zip!/3.5_Causal_Inferece/3.5_Causal_Chat5.pdf]

Combining statistical and causal thinking_5 - Clear memory request

Please clear memory.

I've cleared the memory of all stored information about you. Let me know how I can assist you going forward!

Take the role of an expert biostatistician for this Chat

Understood. I'll take the role of an expert biostatistician for this chat. Let me know what you need help with—study design, causal inference, survival analysis, missing data, or anything else within the field.

I have data from a randomised clinical trial. I want to compare the efficacy of the experimental treatment with that of the control on overall survival in patients with non-small cell lung cancer. In this two-arm RCT, a considerable number of patients in the control arm switched to the experimental treatment after disease progression, while those in the experimental arm never switched.
My goal is to estimate the causal effect of sustained intake of the experimental treatment versus sustained intake of control on Overall survival, had the control group patients not been allowed to switch. Based on the information provided above, please define my target Estimand, following the guidance from the Estimand framework (ICH-E9 addendum).

To define your target estimand following the **ICH E9(R1) Addendum on Estimands and Sensitivity Analysis**, we need to specify its five attributes: **treatment**, **population**, **variable (endpoint)**, **intercurrent events**, and **summary measure**.

🔹 Target Estimand Definition

1. Treatment:

- Comparison of two strategies:
  - **Experimental arm**: Sustained administration of the experimental treatment from randomisation.
  - **Control arm**: Sustained administration of the control treatment from randomisation, **had no switching to the experimental treatment occurred**.

2. Population:

- All randomised patients with non-small cell lung cancer in the intent-to-treat (ITT) population.

3. Variable (Endpoint):

- **Overall survival (OS)**, defined as the time from randomisation to death from any cause.

4. Intercurrent Events and Strategy:

- **Treatment switching** in the control arm (from control to experimental treatment after progression) is handled using a **hypothetical strategy**, i.e. we aim to estimate what OS would have been **had no switching occurred**.
- Other intercurrent events (e.g., loss to follow-up, non-adherence) should be addressed based on their nature. Commonly:
  - **Loss to follow-up**: Treated as **censoring**, assuming non-informative censoring or handled via appropriate causal methods if informative.
  - **Non-adherence** in the experimental arm is not relevant if your hypothetical strategy assumes full adherence.

5. Summary Measure:

- Difference in survival curves, hazard ratio, or restricted mean survival time (RMST) comparing the two treatment strategies, under the hypothetical scenario of no switching in the control arm.

✅ Formal Estimand Statement (ICH E9 Format)

> **The estimand** targets the difference in overall survival between randomised patients receiving sustained experimental treatment versus sustained control treatment, had control patients not been allowed to switch to the experimental arm after progression. The population comprises all randomised patients with non-small cell lung cancer. The variable is overall survival from randomisation to death from any cause. The intercurrent event of treatment switching in the control arm is addressed using a **hypothetical strategy**, imagining a scenario where no switching occurred. The summary measure is the hazard ratio (HR) comparing the two strategies.

Let me know if you'd like help selecting appropriate estimation methods (e.g. IPCW, RPSFTM, two-stage estimator, cloning methods), or if you want to define a **supporting estimand** for sensitivity analysis.

The switching behaviour is affected by a set of different baseline and time-varying covariates. Here, I want to assume that the time-varying confounders that influence the switching behaviour and Overall survival are also themselves affected by prior treatment intake.
For e.g. ECOG score might not only affect whether patients should switch treatment or not, but its value might also be affected by prior treatment intake reflected by the switching behaviour.
In this case, the analytical problem becomes more complex. Can you explain?

Yes, you're describing a **classical case of time-varying confounding affected by prior treatment**, which introduces **treatment–confounder feedback**. This complicates the analysis and **invalidates standard methods** for causal estimation (like Cox regression with time-varying covariates or standard inverse probability of treatment weighting).

Let me break down the problem and its implications:

🔍 1. What Is Happening in Your Setting?

- Patients in the **control arm** may **switch to the experimental treatment** after disease progression or other clinical indications.
- **Switching is not random**: it depends on **covariates**, including **time-varying factors** like:
    - Disease progression status
    - ECOG performance score
    - Target lesion size
- These **time-varying covariates both**:
    - Affect the **decision to switch** (they are **confounders**),
    - Are affected by **prior treatment** (they are **intermediate variables**).

➡️ This leads to **treatment–confounder feedback**.

🔁 2. Why This Is a Problem for Standard Methods

📉 Standard time-varying Cox models or naïve ITT/PP approaches:

- **Adjusting for post-baseline confounders directly** (e.g. in a time-dependent Cox model) would **block** part of the causal pathway from treatment to outcome, **biasing the estimate** downward (collider bias or overcontrol).
- **Not adjusting for them** leads to **confounding bias**, since switching is informative.

🧮 IPCW (Inverse Probability of Censoring Weighting) issues:

- IPCW can be used under a **hypothetical strategy** by censoring patients at switching and reweighting to adjust for selection bias.
- However, **standard IPCW** will **fail** if time-varying covariates affected by prior treatment are used to predict switching, as this introduces **bias due to treatment–confounder feedback**.

🧠 3. Conceptual Framework: Dynamic Treatment Regimes and g-methods

To properly estimate the **causal effect of sustained treatment strategies**, you must use methods that **appropriately account for time-varying confounding affected by prior treatment**:

✅ Suitable methods include:

- **Marginal Structural Models (MSMs)** with **IPTW of the treatment** (not censoring!)

- **G-computation / Sequential g-formula**
- **G-estimation of Structural Nested Models (SNMs)**

These methods:

- Model the **time-varying nature of treatment and covariates**,
- **Separate direct and indirect pathways** of treatment influence,
- Allow estimation of the causal effect under **sustained treatment strategies** (e.g. never switch vs. always switch).

🧭 4. Causal DAG Illustration (simplified)

```scss
    A(t-1) ──▶ L(t) ──▶ A(t)
        │          │
        ▼          ▼
       Y(t) ◀──────
```

Where:

- `A(t)` = treatment at time t (including switching),
- `L(t)` = time-varying covariates at time t (e.g., ECOG),
- `Y(t)` = survival outcome.

Here:

- `L(t)` is a **confounder** of A(t) → Y(t),
- but also a **mediator** of A(t-1) → Y(t),
- which violates standard conditional exchangeability assumptions if not handled correctly.

📌 Summary

Because time-varying covariates are **both confounders and mediators**, you need:

- **g-methods** that properly handle **treatment–confounder feedback**,
- clear specification of your **target estimand under a hypothetical strategy**,
- careful implementation of estimation techniques (e.g. MSM with IPTW of treatment).

Let me know if you want help specifying the model, implementing it in code (e.g. R or Stata), or validating assumptions (e.g. positivity, correct model specification, no unmeasured confounding).

Depict our research problem in a DAG

Here is a **Directed Acyclic Graph (DAG)** that represents your research problem:

🎯 Causal DAG for Treatment Switching in a Two-Arm RCT with Time-Varying Confounding

We focus on:

- **Treatment assignment** at baseline (A_0): Experimental vs. Control (randomised)
- **Time-varying treatment (A_1, A_2,...)** in the control arm due to switching
- **Time-varying covariates (L_1, L_2,...)** such as ECOG score, progression
- **Outcome (Y)**: Overall survival
- **Feedback loop**: Prior treatment influences covariates, which influence future treatment

---

🧭 DAG Structure (simplified for 2 time points)

```
plaintext

          A₀
         /  \
       ↓      ↓
     L₁        A₁
      ↓       /  \
      ↓      ↓     ↓
      Y     L₂ ←——
              ↓
              Y
```

🔍 Explanation of Nodes and Arrows

Node	Meaning
A_0	Randomised treatment at baseline (experimental vs. control)
L_1	Time-varying covariate at time 1 (e.g., ECOG, progression)
A_1	Treatment status at time 1 (e.g., switched or not)
L_2	Time-varying covariate at time 2
Y	Survival outcome (overall survival)

🔄 Key Features of the DAG

- $A_0 \rightarrow L_1 \rightarrow A_1$: Treatment at baseline affects future covariates, which affect the switching decision
- $A_1 \rightarrow L_2 \rightarrow Y$: Post-switch treatment affects later covariates and ultimately survival
- $L_1 \rightarrow Y$: Early covariates also directly affect outcome
- **Feedback loop**: Prior treatment influences covariates, which influence future treatment—**treatment–confounder feedback**
- Although A_0 is randomised, **post-baseline treatment (A_1) is confounded by time-varying variables influenced by prior treatment**

⚠️ Implications for Analysis

- **Standard Cox models adjusting for `L₁`, `L₂`** block part of the causal effect.
- **Not adjusting** leads to **time-varying confounding** bias.
- Use **g-methods** that can handle this structure (e.g., MSMs with IPTW of treatment, g-formula, or SNMs).

Would you like this DAG as a publication-quality figure (e.g. drawn in `dagitty` or `ggdag`) or extended to more time points?

I would like to apply the inverse probability of censoring weighting (IPCW) approach to address the bias introduced by switching following the methodology discussed in the following paper: Robins JM, Finkelstein DM. Correcting for noncompliance and dependent censoring in an AIDS clinical trial with inverse probability of censoring weighted (IPCW) log-rank tests. Biometrics. 2000;56(3):779–88.
Briefly summarise the key points from this paper

Certainly. The foundational paper by **Robins and Finkelstein (2000)** introduced the **Inverse Probability of Censoring Weighting (IPCW)** method to estimate causal treatment effects in the presence of **noncompliance and dependent censoring**, particularly when **post-randomisation events influence subsequent treatment and dropout**.

Here is a **brief summary of the key methodological contributions and insights** from the paper:

📌 Key Points from Robins & Finkelstein (2000):

1. Context and Problem

- The paper addresses causal inference in randomised clinical trials with **noncompliance** (e.g. treatment switching) and **informative censoring** (i.e. censoring depends on post-randomisation variables related to prognosis).
- The motivating example is an **AIDS clinical trial**, but the framework applies to oncology and other settings with treatment switching.

---

2. Main Idea: IPCW to Create a Hypothetical World Without Switching

- Patients who **switch treatments** (or are otherwise noncompliant) are **censored at the time of deviation**.
- To correct for the resulting **selection bias**, each patient is **weighted by the inverse of their estimated probability of remaining uncensored** (i.e., still following the assigned treatment) up to that time.

- This yields a **pseudo-population** in which censoring is **independent** of prognosis (i.e. non-informative), conditional on baseline and time-varying covariates.

---

3. Weight Construction

- Weights are based on **fitted models (e.g. Cox models or pooled logistic regression)** for the probability of remaining uncensored, conditional on observed covariates and treatment history.
- Denote censoring indicator by $C(t)$, and define the weight as:

$$w_i(t) = \prod_{s \leq t} \frac{1}{\Pr(C_i(s) = 0 \mid \bar{L}_i(s), \bar{A}_i(s), \bar{C}_i(s-1) = 0)}$$

  where $\bar{L}_i(s)$, $\bar{A}_i(s)$ denote the covariate and treatment history up to time s.

---

4. Application: Weighted Log-Rank Test and Survival Curves

- The IPCW weights are used in **weighted versions of the log-rank test** and **Kaplan-Meier estimators**, allowing consistent estimation of survival under a **hypothetical intervention** where patients had followed their assigned treatment throughout.
- These estimators are **unbiased under the assumption of no unmeasured confounding** and correct model specification for the censoring mechanism.

---

5. Assumptions

- **Positivity**: Every patient has a non-zero probability of not switching at each time point given their covariate history.
- **No unmeasured confounding** of the censoring process (i.e., all predictors of switching and survival must be measured and included in the model).
- **Correct model specification** of the censoring model.

---

6. Advantages

- Avoids structural model assumptions (unlike structural nested models or rank-preserving structural failure time models).
- Easily interpretable: estimates survival curves or log-rank comparisons under the **hypothetical no-switching scenario**.
- Applicable to trials where **treatment switching is affected by post-randomisation variables**.

---

7. Limitations and Sensitivity

- Sensitive to **misspecification** of the censoring model.

- Weights can be unstable if **predicted probabilities are very small** (→ use of **stabilised weights** or **weight truncation** recommended).
- Assumes **correct identification of covariates driving switching and survival**.

🧠 Summary

> Robins and Finkelstein (2000) propose using **IPCW** to estimate survival under a hypothetical strategy where patients **fully adhere to their randomised treatment**. The method handles **time-varying covariates affected by prior treatment**, avoids biases from informative censoring due to switching, and enables valid causal inference **without structural model assumptions**—provided all relevant covariates are measured and correctly modelled.

Let me know if you want help applying IPCW in your setting (e.g. model specification, weight diagnostics, code in R), or if you'd like a visual to illustrate how the IPCW pseudo-population works.

Could you elaborate more on the research question Robins and Finkelstein were trying to answer in their paper, mentioning the comparator arms, possible intercurrent events, outcome definition and population under study.

Certainly. Robins and Finkelstein (2000) sought to address a causal research question in the context of an AIDS clinical trial. Here's a structured breakdown of their **research question** framed in terms consistent with modern causal inference and the **estimand framework** (ICH E9(R1)):

🎯 Research Question in the Original Paper

> *What is the effect of being assigned to receive immediate versus deferred zidovudine (AZT) treatment on overall survival, under a hypothetical scenario in which patients adhered to their randomised treatment throughout follow-up, despite observed switching or noncompliance?*

🔬 Study Context

- **Trial**: The motivating example is based on the **AIDS Clinical Trials Group Study 019** (ACTG 019), which randomised patients to **immediate** vs. **deferred** zidovudine treatment.
- **Intervention (Treatment Arm)**: Start zidovudine (AZT) immediately upon trial entry.
- **Comparator (Control Arm)**: Delay zidovudine until the CD4 count drops below a predefined threshold.

👥 Population

- HIV-positive patients enrolled in the ACTG 019 trial.
- Inclusion criteria: patients with CD4 cell counts above a certain level at baseline and no AIDS-defining illness at entry.

- The analysis was performed on the **intention-to-treat (ITT)** population, but the target estimand deviated from the standard ITT effect.

📉 Variable (Endpoint)

- **Overall survival**, defined as the time from randomisation to **death from any cause**.

⚠️ Intercurrent Events

- The major **intercurrent event** was **noncompliance or switching**:
  - Patients in the **deferred arm** often **initiated AZT earlier than prescribed** (i.e., before meeting the CD4 threshold).
  - Patients in the **immediate arm** might **discontinue treatment**.
- These intercurrent events were **not random**: they were associated with **post-randomisation factors** like CD4 counts or clinical deterioration, which were also predictive of death.

🎯 Estimand Targeted in the Paper

Using modern language:

Component	Description
**Treatment**	Immediate AZT vs. deferred AZT as randomised
**Population**	All randomised patients in ACTG 019
**Variable**	Time from randomisation to death (overall survival)
**Intercurrent events**	**Noncompliance/switching** was handled via a **hypothetical strategy**, where patients are assumed to have remained on their assigned treatment throughout
**Summary measure**	Causal effect on survival curves or hazard ratio under a hypothetical no-switching world (via weighted log-rank tests and survival estimation)

🧠 Key Methodological Challenge

- Standard ITT analysis is **biased toward the null** due to **treatment crossover** (e.g., early AZT initiation in the deferred arm).
- Per-protocol or as-treated analyses would also be biased because **treatment changes depend on prognostic covariates** (e.g., declining CD4 count).
- Time-varying covariates like CD4 count are **confounders of treatment and survival**, and are **themselves influenced by prior treatment** — leading to **treatment–confounder feedback**.

🧪 Solution Proposed

- **Censor patients at time of switching**, and use **inverse probability of censoring weighting (IPCW)** to correct for the **selection bias** this introduces.

- This allows estimation of **survival under a hypothetical scenario** where patients remained on their assigned treatment.

📌 In Summary

Robins and Finkelstein addressed a problem of **estimating the causal effect of treatment assignment in the presence of non-random switching**, using IPCW to reconstruct what survival would have looked like **if patients had adhered to their initial treatment assignment**. Their approach laid the groundwork for many causal methods used today to handle **intercurrent events** like **treatment switching**, particularly in trials where switching is influenced by time-varying clinical status.

Let me know if you'd like help translating these ideas into your trial's setting (e.g. constructing weights, defining the hypothetical strategy more precisely, or comparing to other methods like RPSFTM or MSMs).

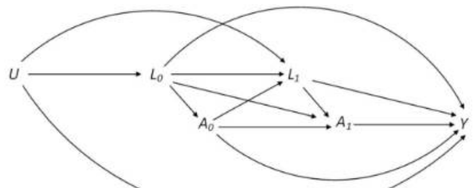

Fig. 1 Graphical representations of our research focus and the bias of naive methods for time-varying confounding in the presence of treatment-confounder feedback: This Directed acyclic graph (DAG) represents the post-progression period for patients in the control arm. Following disease progression, the decision as to whether or not a patient in the control group continues to take its treatment (A_0) or (A_1) depends on predictive factors measured at or before the time of disease progression (L_0). Again, we assume that treatment intake (A_0) affects outcome (Y) and influences a set of TVCs at a later time-point (L_1). These covariates are also assumed to impact the clinician's decision as to whether or not a patient should subsequently change treatments (A_1) introducing time-varying confounding and treatment-confounder feedback through L_1

Attached, I provide you with a directed acyclic graph (DAG) that represents our research problem.
To simplify this DAG and take into consideration this deterministic information provided below, I restrict my DAG to the post-progression period for patients in the control arm only and consider two follow-up time points, 1 & 2.
Here A(k) is deterministically 0 if A(k-1)=0 AND L(k-1) (reflecting progression status)=0 (no progression).
Following disease progression, the decision as to whether or not a patient in the control group continues to take their treatment (A0 or A1) depends on predictive factors measured at or before the time of disease progression (L0).
Again, we assume that treatment intake (A0) affects outcome (Y) and influences a set of TVCS at a later time-point (L1).
These covariates are also assumed to impact the clinician's decision as to whether or not a patient should subsequently change treatments (A1), introducing time-varying confounding and treatment confounder feedback through L1.
I will emphasise again that in our case scenario, patients in the control group do not switch unless they progress, and all patients in the experimental group never switch.
I still want to estimate in my outcome model the causal effects of sustained treatment interventions (always taking control versus always taking experimental) under a hypothetical strategy where control patients do not switch treatment.
I want you to use this DAG as a basis for performing the appropriate IPCW analyses and provide me with the analysis R-script.
You may assume that the dataset is in long format, with variables: id, time (in months and increases

with increments of 1), death indicator, switching indicator, baseline covariates repeated over time points, time-varying covariates at each time pt.

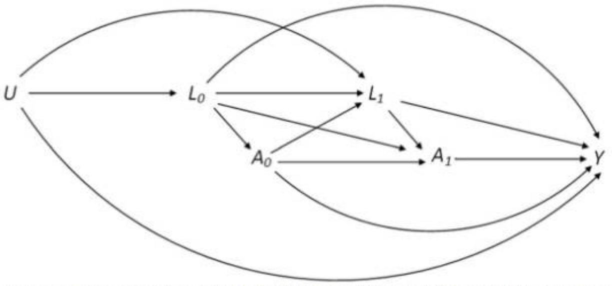

Fig. 1 Graphical representations of our research focus and the bias of naive methods for time-varying confounding in the presence of treatment-confounder feedback: This Directed acyclic graph (DAG) represents the post-progression period for patients in the control arm. Following disease progression, the decision as to whether or not a patient in the control group continues to take its treatment (A_0) or (A_1) depends on predictive factors measured at or before the time of disease progression (L_0). Again, we assume that treatment intake (A_0) affects outcome (Y) and influences a set of TVCs at a later time-point (L_1). These covariates are also assumed to impact the clinician's decision as to whether or not a patient should subsequently change treatments (A_1) introducing time-varying confounding and treatment-confounder feedback through L_1

Thank you. Based on your DAG and the clarified data structure and assumptions, here is how we can approach the **IPCW analysis** in R to estimate the **causal effect of sustained treatment** (experimental vs. control) on overall survival under a **hypothetical no-switching strategy**.

⚙️ IPCW Setup Based on Your DAG

You assume:

- Switching can only occur after progression.
- Treatment status is **deterministically** 0 before progression.
- Time-varying confounders (`L1`) are affected by prior treatment (`A0`) and in turn affect future treatment decisions (`A1`) and survival (`Y`).

📦 Key Variables in Long Format

Variable	Description
`id`	patient identifier
`time`	follow-up month (0, 1, 2, …)
`death`	1 = died at this time, 0 = otherwise
`switched`	1 = switched to experimental at this time, 0 = not switched
`A`	treatment status at time `t` (1 = experimental, 0 = control)
`L0`, `L1`	baseline and time-varying covariates (e.g., ECOG, progression)
`censor`	1 = censored due to switching (only for control group)

✅ Goal

Censor patients at switching, then apply **IPCW** to reweight those who remain uncensored, simulating a world where **no switching occurs**.

📜 R Script for IPCW Analysis

```r
# Load required packages
library(survival)
library(ipw)
library(dplyr)

# Assumed dataset: `data_long` in long format
# Variables: id, time, death, switched, A, L0, L1, censor

# Step 1: Define censoring variable
# Censor at the time of switching (only for control patients)
data_long <- data_long %>%
  group_by(id) %>%
  mutate(censor = ifelse(switched == 1 & A == 0, 1, 0)) %>%
  ungroup()

# Step 2: Fit model for probability of remaining uncensored (treatment-specific model)
# Only control patients are eligible for censoring
model_censor <- glm(censor ~ time + A + L0 + L1,
                    family = binomial(), data = data_long)

# Step 3: Predict probability of remaining uncensored
data_long <- data_long %>%
  mutate(
    p_uncens = 1 - predict(model_censor, type = "response"),
    weight_uncens = 1 / p_uncens
  )

# Optional: truncate extreme weights
data_long <- data_long %>%
  mutate(weight_uncens = pmin(pmax(weight_uncens, 0.01), 10))

# Step 4: Collapse to one row per patient for survival analysis
# Assuming last record per patient has event or censoring info
data_surv <- data_long %>%
  group_by(id) %>%
  summarise(
    time = max(time),
    death = death[which.max(time)],
    A0 = A[which.min(time)],
    weight = weight_uncens[which.max(time)]
  )

# Step 5: Weighted survival analysis using weighted Cox model
cox_ipcw <- coxph(Surv(time, death) ~ A0, weights = weight, data = data_surv)
summary(cox_ipcw)

# Optional: Plot weighted survival curves
library(survminer)
fit_km <- survfit(Surv(time, death) ~ A0, weights = weight, data = data_surv)
ggsurvplot(fit_km, data = data_surv, conf.int = TRUE, pval = TRUE)
```

📌 Notes

- If you have multiple time-varying covariates (`` `L1` ``, `` `L2` ``, etc.), update the model accordingly.
- This script estimates survival under a **hypothetical strategy where switching is prohibited**, by censoring at switch time and reweighting to account for dependent censoring.
- If you want to estimate **restricted mean survival time (RMST)** or other contrasts, let me know.

Would you like help extending this to **multi-time-point** or **MSM**-based IPCW instead of censoring-based IPCW?
